# The Effect of Dietary Prebiotics and Probiotics on Body Weight, Large Intestine Indices, and Fecal Bile Acid Profile in Wild Type and IL10−/− Mice

**DOI:** 10.1371/journal.pone.0060270

**Published:** 2013-03-21

**Authors:** Shiu-Ming Kuo, Patricia M. Merhige, Lee R. Hagey

**Affiliations:** 1 Department of Exercise and Nutrition Sciences, University at Buffalo, Buffalo, New York, United States of America; 2 Division of Gastroenterology, Department of Medicine, University of California San Diego, San Diego, California, United States of America; Wageningen University, The Netherlands

## Abstract

Previous studies have suggested roles of probiotics and prebiotics on body weight management and intestinal function. Here, the effects of a dietary prebiotic, inulin (50 mg/g diet), and probiotic, *Bfidobacterium animalis* subsp. *lactis* (Bb12) (final dose verified at 10^5^ colony forming unit (cfu)/g diet, comparable to human consumption), were determined separately and in combination in mice using cellulose-based AIN-93G diets under conditions allowed for the growth of commensal bacteria. Continuous consumption of Bb12 and/or inulin did not affect food intake or body, liver, and spleen weights of young and adult mice. Fecal bile acid profiles were determined by nanoESI-MS/MS tandem mass spectrometry. In the presence of inulin, more bacterial deconjugation of taurine from primary bile acids was observed along with an increased cecal weight. Consumption of inulin in the absence or presence of Bb12 also increased the villus cell height in the proximal colon along with a trend of higher bile acid sulfation by intestinal cells. Feeding Bb12 alone at the physiological dose did not affect bile acid deconjugation and had little effect on other intestinal indices. Although interleukin (IL)10-null mice are susceptible to enterocolitis, they maintained the same body weight as the wild type mice under our specific pathogen-free housing condition and showed no signs of inflammation. Nevertheless, they had smaller cecum suggesting a mildly compromised intestinal development even before the disease manifestation. Our results are consistent with the notion that dietary factors such as prebiotics play important roles in the growth of intestinal microbiota and may impact on the intestinal health. In addition, fecal bile acid profiling could potentially be a non-invasive tool in monitoring the intestinal environment.

## Introduction

The symbiotic relationship between the host and intestinal microbiota has been extensively studied, in part because of its implications in intestinal health [1]–[3]. To promote the development of beneficial microbiota in the intestine, prebiotics and probiotics such as inulin (fructooligosaccharide) and Bb12 (*Bifidobacterium animalis* subsp. *lactis*) have been added separately or in combination to human food items. Inulin is digested mainly by cecal microbiota after passing through small intestine [4], [5], and was shown to stimulate the growth of bifidobacteria consistently [6]–[10]. The probiotic Bb12 was shown to tolerate the low pH found in the stomach [11], [12] and be present in the content of proximal colon after feeding [13]. Various carbohydrate sources including inulin are known to support the growth of Bb12 [11].

The mouse model was used in this study for several reasons. One area of interest was the proposed contrasting effects of pre- and probiotics on energy balance. Intestinal microbiota has been implicated as a potential cause of the global obesity epidemic although the mechanism was not clear [14], [15]. Yet, dietary inulin and other soluble fibers have been found to promote short-term satiety and thus could reduce caloric intake [16], [17]. The first aim of the study was to determine whether pre- and probiotics affect body weight, organ weight, and intestinal histology in young and adult mice under *ad libitum* feeding condition. Animal studies allow for the preparation of isocaloric pre- and probiotics-containing diets. Furthermore, continuous monitoring of food intake and body weight is possible in animal models, which helps to address long-term effect, if any, on body weight and organ development.

Mouse model also permits a comparison between wild type and interleukin (IL)10-null mice in their responses to the pre- and probiotic feeding, and the wildtype to IL10-null comparison is a subpart of the Aim 1. The loss of the anti-inflammatory cytokine IL10 in mice increased their susceptibility to intestinal inflammation and led to weight loss when housed in an environment that was not pathogen-free [18]–[20]. However, it is not clear whether the loss of IL10 has impact on the intestinal epithelium in the absence of pathogens. Specifically, whether the loss of IL10 affects the response to the feeding of pre- and probiotics, conditions that could affect commensal bacteria. A three-way talk between the immune system, commensal bacteria and intestinal epithelial cells is known [21], [22]. Because some commensal bacteria have been shown to exert biological effect through modulating the IL10 expression of intestinal T-cells [23], the loss of IL10 may affect the host response to pre- and probiotics.

The effect of pre- and probiotics on bile acid metabolism is the second Aim and it has several implications. Dietary prebiotics were found not to affect total bile acid pool size in rats [24] but it is not clear whether prebiotics and/or probiotics affect bile acid metabolism. Fecal bile acid profiling performed here reflects the sum of host hepatic and intestinal metabolism as well as intestinal microbial activity. It is important to characterize changes in bile acid metabolism partly because primary bile acids were known to affect intestinal water secretion [25], [26]. Our results should also provide information on the feasibility of using the non-invasive fecal bile acid profile as a biomarker for the intake of pre- and probiotics. While species difference in the structure of primary taurine-conjugated bile acids from the liver is known [27], in the intestinal environment primary taurine-conjugated bile acids from various species are similarly subjected to the bacteria-mediated deconjugation [28]. Such deconjugation activity was demonstrated in Bb12 [12]. After deconjugation, taurine cannot be added back to bile acids by bacteria or intestinal cells. As a result, taurine conjugate in feces represents the leftover primary bile acid and more intestinal bacterial activity is expected to lead to less leftover taurine conjugate. While little primary bile acid is present in the sulfate form, a proportion of the deconjugated secondary bile acids in the intestine can become sulfate-conjugated probably by the activity of the intestinal cells [29]. Because of the similarity in bile acid metabolism, mouse model has been used widely to understand factors influencing human bile acid metabolism [30]–[32]. The use of mouse model also allowed a comparison between wild type and IL10-null mice on bile acid metabolism in the intestine, which was also not previously examined and it was a subpart of the Aim 2. A change in the bile acid metabolism in the intestine due to IL10 gene knockout would be consistent with a compromised intestinal environment in these mice.

## Materials and Methods

### IL10 knockout mouse colony

The transgenic IL10 knockout mouse colony maintenance and all animal handling protocols were approved by the Institutional Animal Care and Use Committee of the University at Buffalo (protocol #NUT06015N and #PTE18116N). Specific pathogen-free (SPF) [33] wild type C57BL/6 male mice were purchased from Taconic Farms, Inc. (Germantown, New York). The male IL10+/+ (wild type) and IL10−/− mice were generated by heterozygous inbreeding from our IL10-null colony [34]. Whenever possible, littermates were assigned to different dietary groups. Genotyping to determine IL10+/+ (wild type) and IL10−/− was performed by the PCR analysis of DNA extracted from ear pieces using three primers: IL10T 1.4: GCCTTCAGTATAAAAGGG GGA CC; IL10 T2.2: GTGGGTGCAGTTATTGTC TTCCCG; IL10 NEO: CCTGCGTGCAATCCATCTTG. The 200 bp PCR product from the first two primers indicates the presence of wild type allele, and the 450 bp PCR product from the 2nd and 3rd primers indicates the presence of knockout allele. The IL10 null genotype of the colony was confirmed by a heightened sensitivity to lipopolysaccharide-induced inflammatory response in our periodical tests (results not shown). While in the SPF colony, the mice were given an irradiated NIH31 diet (Harlan Laboratories, Teklad diet 7913) and autoclaved water. They were moved to the conventional animal facility immediately before the feeding study.

### Experimental diet formulation and storage

Four AIN93G-based experimental mouse diets (Table 1) were prepared and pelleted by Dyets Inc. (Bethlehem, PA). The food-grade inulin was from chicory roots (Frutafit HD from Sensus, The Netherlands). The food-grade probiotics, Bb12 (5.2×10^11^ colon forming unit (cfu)/g), was from Chr. Hansen Inc. (Milwaukee, WI). To ensure that all diets were isocaloric, the amount of dextrose (Dyetrose) and cellulose were adjusted in the inulin-supplemented diet (Table 1). To determine the physiological relevant dose of Bb12 in the mouse diet, several factors were taken into the consideration: the effective, well-tolerated and physiologically relevant dose of Bb12 in human studies was 10^9^–10^10^ cfu/day [35]; the body size difference between human and mice is roughly 10,000 fold; and food intake is 3–4 g/mouse day. As a result, Bb12 concentration of 10^5^ cfu/g diet pellet was planned for the mice. Because the room-temperature (<27°C) extrusion procedure during the pellet preparation involved the addition of small amount of water, a potential loss of Bb12 viability during extrusion was monitored. Furthermore, the viability of Bb12 under different storage conditions was validated. The purpose was to ensure that mouse diets contained the same level of viable Bb12 throughout the duration of the study. The Bb12 viability analysis [36] was performed by individuals blinded to the diet formulation. All four diets were prepared at the same day. To avoid Bb12 residues in the non-probiotic diet, the diets without Bb12 supplementation, Control and Inulin, were prepared first. Other publications have conducted pre- and probiotic supplementation by including them in the diet [37]–[40].

**Table 1:** Composition and mixing sequence of AIN-93G-based experimental diets.

	Diet			
	Control	Inulin	Bb12	Inulin+Bb12
**Ingredient**	**g/kg diet**			
Casein	160	160	160	160
Sucrose	100	100	100	100
Soybean Oil	70	70	70	70
t-Butylhydroquinone	0.014	0.014	0.014	0.014
Dyetrose	132	104	132	104
Cellulose	50	28	50	28
Inulin	0	50	0	50
Cornstarch	397.5	397.5	396.5	396.5
**Premix 1**	**g/kg diet**			
AIN-93 Mineral Mix	35	35	35	35
AIN-93 Vitamin Mix	10	10	10	10
L-Cystine	3	3	3	3
Choline Bitartrate	2.5	2.5	2.5	2.5
**Premix 2**	**g/kg diet**			
Casein	40	40	40	40
Bb12 (5.2×10^11^ cfu/g)	0	0	1	1
Kcal/kg diet	3760	3760	3756	3756

### Feeding studies

**Figure 1:**
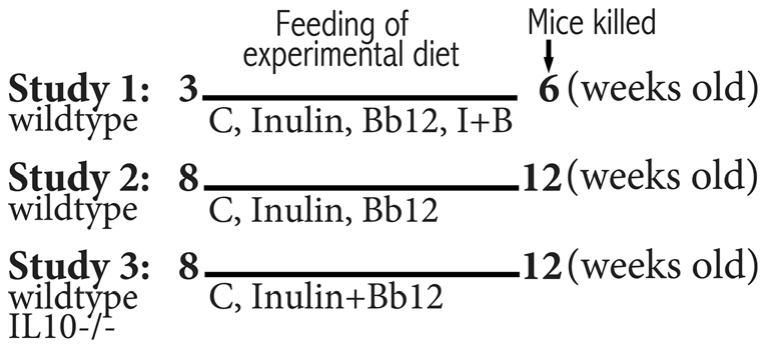
The mouse strain, starting age and feeding condition of three studies.

Mice were housed individually for the feeding study. Diet intake and body weight were monitored weekly. The mouse strain, starting age of mice and the feeding condition of the three studies are shown in Fig. 1. Study 1 used 4 males per group immediately after weaning. Study 2 and Study 3 had 6 males and 9–14 males per group, respectively. Mice in Study 2 and Study 3 were acclimated to individual housing and control AIN-93G diet for a week before assigned to different diet groups. After the 3–4 weeks of feeding, mice were killed at 4–6 hours after the start of the light cycle by cervical dislocation and then decapitated for blood collection. The plasma was used for the IL6 measurement by ELISA following manufacturer's instructions (eBioscience, catalog #88-7064). Organs were harvested for wet weight measurement. Cecum wet weight included the content. Fresh fecal samples (2–3 pellets/mouse) were stored at −80°C until bile acid analysis. Also, a 2 mm segment of the proximal colon (about 10 mm below cecum) was collected from each mouse for paraffin embedding by the University at Buffalo Histological Services core facility. All hematoxylin and eosin-stained colon sections were analyzed by a single person blinded to the dietary treatment and genotype. The mean cell height of up to 10 measurements of each cell type was used as the representative value of a mouse. A figure illustrating various histological areas used for the cell type analysis is included in the supplemental material (Fig. S1).

### Bile acid analysis

Bile acid analysis was performed following a published nanoESI-MS/MS tandem mass spectrometry method [29]. Briefly, bile acids were extracted from the fecal pellets by dissolved in reagent-grade methanol/1% isopropanol and analyzed using a Perkin Elmer Sciex API-III instrument (Perkin-Elmer, Alberta, Canada) modified with a nanoelectrospray source from Protana A/S (Protana, Odense, Denmark). Palladium-coated borosilicate glass capillaries (Protana) were used for the sample injection. The instrument was operated in the negative mode with ion spray voltage set to 600 V, interface voltage at 110 V and orifice voltage at 90 V. A curtain gas of ultrapure nitrogen was pumped into the interface at 0.6 l/min to aid the evaporation of solvent droplets and to prevent particulate matters from entering the analyzer. The sample was examined in the Q1 mode, and the chemical identity of peaks confirmed by the fragmentation pattern of selected ions (Q3 mode) using argon gas. Sulfate (m/z parents of 97) and taurine (m/z parents of 124) conjugates were identified. For calculation, peak height values obtained by ESI-MS/MS analysis were normalized by the peak height of the dominant trihydroxy bile acid in mice (m/z 407) [29].

### Statistical analysis

For Studies 1 and 2, one-way ANOVA (effect of diet) with post-hoc Student-Newman-Kuels multiple comparison was used. For the third study, two-way ANOVA (effects of diet and genome) was performed along with custom hypothesis tests between groups. None of the two-way ANOVA found interaction between genotype and dietary treatment. In some cases as described in the Table and Figure legends, nonparametric Kolmogorov – Smirnov two sample test [41], [42] was performed. A significant effect was concluded when p<0.05.

## Results and Discussion

### Bb12 viability in the diet

**Figure 2:**
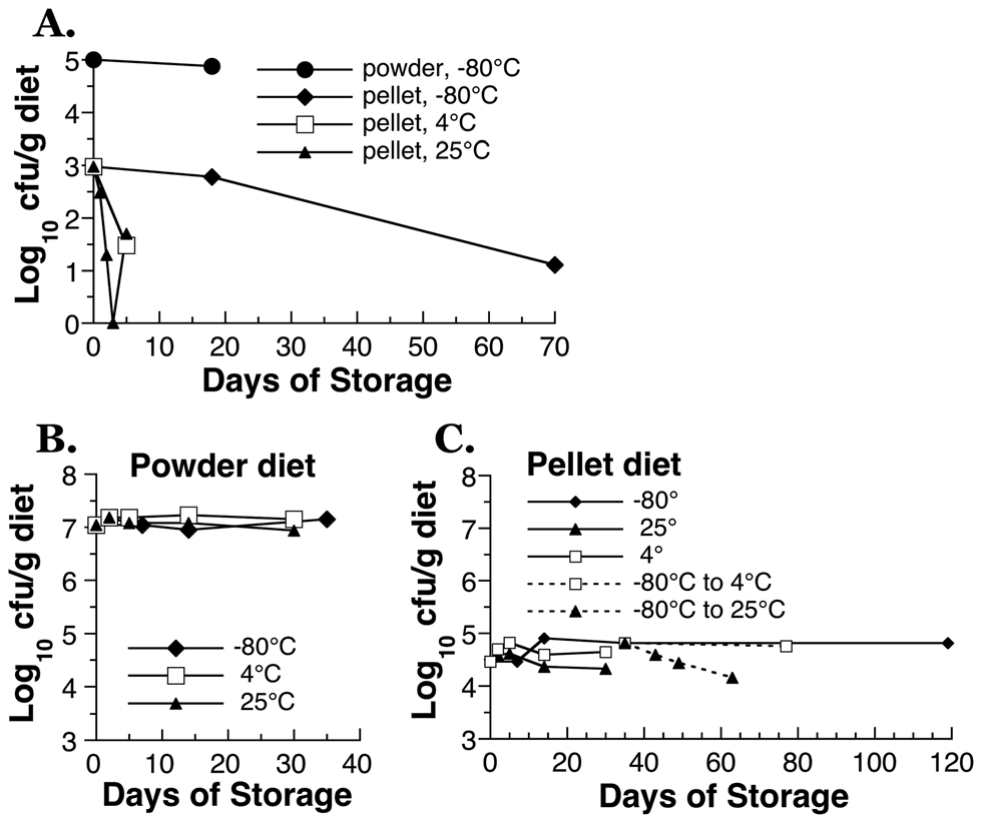
Development and validation of pelleted AIN93G-based mouse diet containing probiotic, Bb12. AIN-93G diet was formulated to deliver Bb12 at (A) 10^5^cfu/g powder diet and (B, C) 10^7^ cfu/g powder diet. The viability of Bb12 was also determined after the power diet was pelleted at room temperature (<27°C); and after diets have been kept under various storage conditions, −80°C, 4°C and 25°C. These storage temperatures mimic the long-term storage, short-term storage and weekly feeding condition we used in three studies. (A) Poor viability of Bb12 in pelleted AIN93G diet when Bb12 was premixed with the vitamin/mineral mix. (B, C) Bb12 was premixed with casein and then used for the preparation of AIN93G diet. Adequate Bb12 viability at −80°C, 4°C and 25°C storage was found in both (B) powder and (C) pellet AIN93G-based diets when premixed with casein. Pellet diet was used for the studies reported.

To ensure that the probiotics, Bb12, was given to mice at the intended dose and form, we first validated the Bb12 in the diet. Bb12 content in a cup of yogurt is around 10^9^ cfu [35]. With the around 10,000-fold difference in body weight between the two species, the target dose of Bb12 for mouse is in the range of 10^5^ cfu/day. To better mimic the human fashion of probiotic consumption and to avoid the potential complication due to repeated gavage [43], [44], we designed the experiment to have live Bb12 formulated into the diet. Furthermore, to be able to measure intake more precisely, all diets were pelleted after the mixing of various ingredients together. However, these two procedures, ingredient mixing and pelleting, may compromise the viability of Bb12 and thus affect the biological activities of Bb12. The viability in the diet was first evaluated through two trial batches of diet formulated to deliver 10^5^ cfu/g (Fig. 2A) and 10^7^ cfu/g (Fig. 2B–C), respectively. As shown in Fig. 2A and 2B, mixing Bb12 into the powdered AIN93G diet did not lead to any loss of Bb12 viability at either concentration. The room temperature (<27°C) pelleting procedure, in contrast, consistently led to a significant loss of Bb12. A powder diet that initially contained 10^5^ cfu/g had only 10^3^ cfu/g left (Fig. 2A), and a powder diet started with 10^7^ cfu/g had only 10^5^ cfu/g left after pelleting (Fig. 2B vs. 2C). The sequence of ingredient addition was proven critical in maintaining adequate long-term viability of Bb12 in pellets. When Bb12 was mixed into the diet by first adding in the vitamin mix, Bb12 viability decreased quickly in the pellet diet even at −80°C storage (Fig. 2A). When the mixing sequence was modified to follow that described in Table 1, long-term viability of Bb12 in pellet diet was observed (Fig. 2C). The four experimental diets used in the study were then made at the same time and all validated for Bb12 activity. To avoid potential Bb12 contamination, the diets without Bb12 supplementation, Control and Inulin, were prepared first. Bb12-containing diets was made by including 1 g stock Bb12 powder (5.2×10^11^ cfu/g stock powder) per 1 kg diet. Bb12-containing diets with and without inulin had similar final Bb12 activity (10^5^ cfu/g) while Control and Inulin diet had only background levels, 10 and 20 cfu/g, respectively. Bb12 in the pellet diet showed no loss of viability during storage at −80°C (Fig. 2C) and thus −80°C freezer was used for long-term diet storage. The diet was removed from −80°C storage to 4°C in monthly batches for the experimental need. Transferring diet from −80°C to 4°C and subsequent storage at 4°C for 40 days did not affect the viability (Fig. 2C). During animal studies, diets were changed weekly and no significant changes in the viability was expected during a week at room temperature based on the observation in Fig. 2C.

In summary, based on the Bb12 viability validation (Fig. 2) and our feeding protocol, live Bb12 were ingested by mice consistently and continuously as part of the diet at the intended dose during the 3–4 weeks of feeding. In addition, the results of validation experiments showed that gavaging, though commonly used in probiotic studies, is not necessary and thus it is possible to avoid the stressful human intervention [43], [44] in the animal study.

### The effect of pre- and probiotic feeding on food intake and body weight

**Figure 3:**
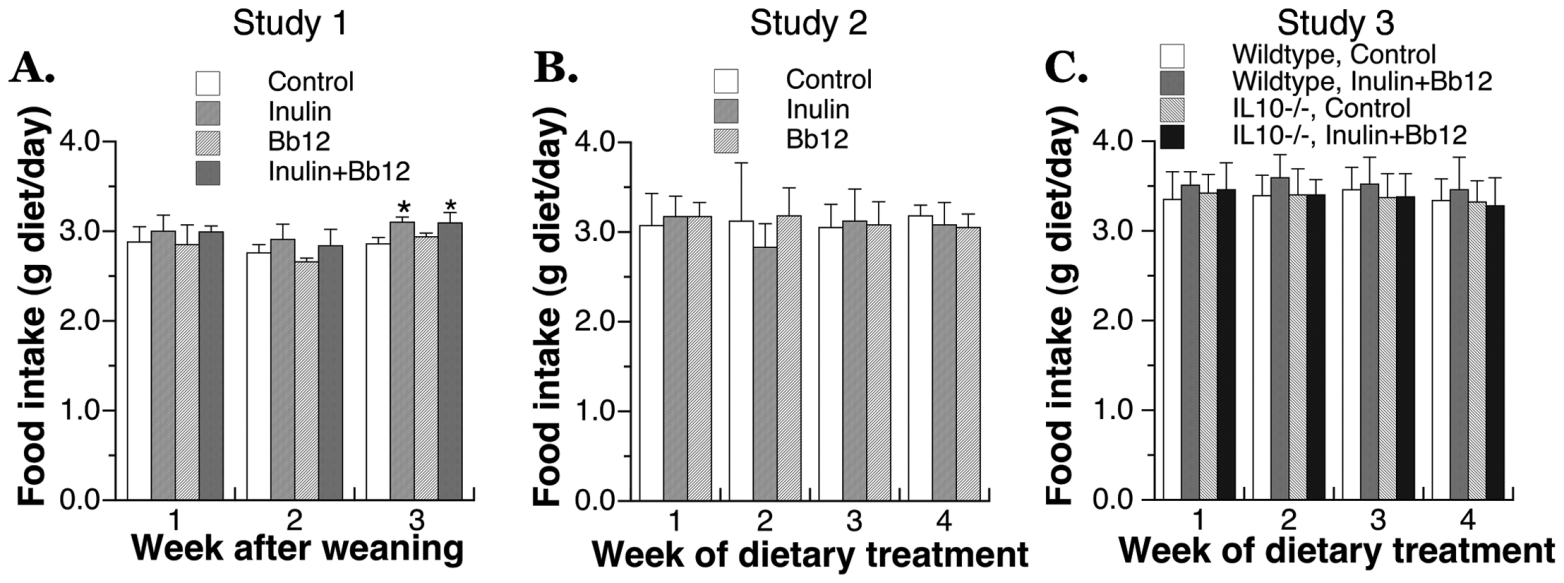
Ad libitum food intake information from three mouse feeding studies using pelleted AIN93G-based experimental diets containing inulin, probiotic Bb12, or inulin+Bb12. (A) Study 1: The 3-week mouse feeding study starting from weaning examining the effect of inulin, Bb12 and their combination (N = 4 in each group); (B) Study 2: The 4-week feeding study examined the effect of inulin and Bb12 on adult mice (N = 6 in each group); (C) Study 3: The effect of 4-week feeding of inulin+Bb12 diet on adult wild type and IL10−/− mice. (N = 9–14). Data are shown as mean±SD. * indicates significantly different from the mice fed the control diet in the same study at p<0.05. There was no genotype-diet interaction found in the 2-way ANOVA performed for Study 3.

**Figure 4:**
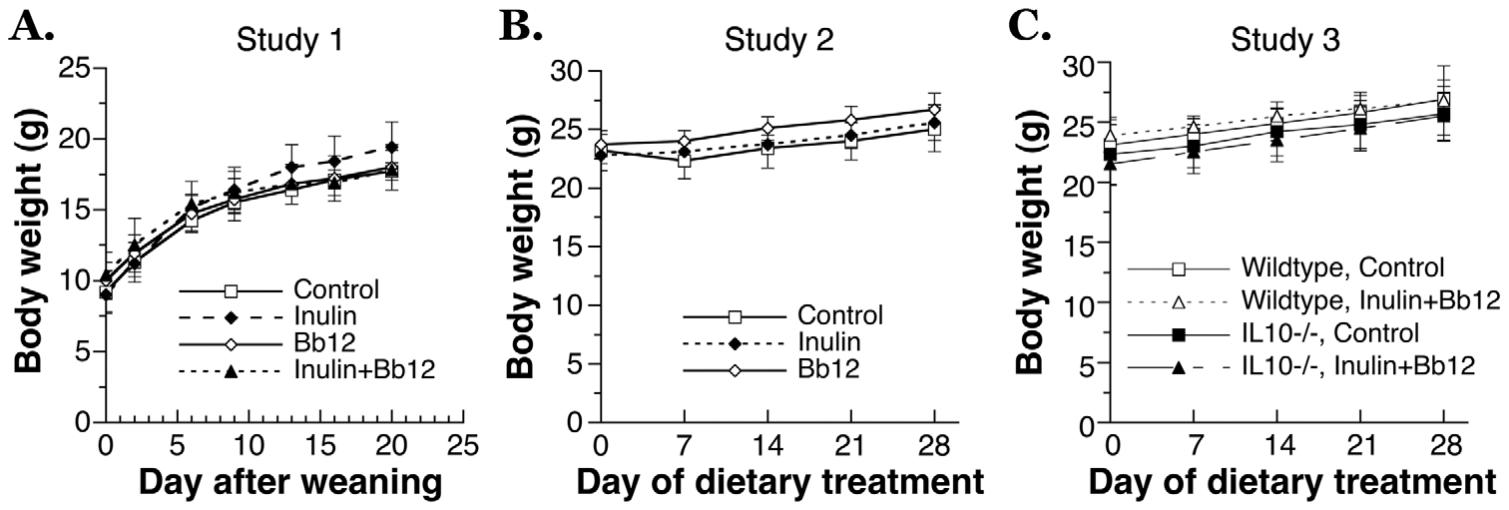
Body weight information from three mouse ad libitum feeding studies using pelleted AIN93G-based experimental diets containing inulin, probiotic Bb12, or inulin+Bb12. (A) Study 1: The 3-week feeding study starting from weaning examining the effect of inulin, Bb12 and their combination (N = 4 in each group); (B) Study 2: The 4-week feeding study examined the effect of inulin and Bb12 on adult mice (N = 6 in each group); (C) Study 3: The effect of 4-week feeding of inulin+Bb12 diet on wild type and IL10−/− mice. (N = 9–14). Data are shown as mean±SD. No significant difference from the control (p<0.05) was observed at any time point. There was no genotype-diet interaction found in the 2-way ANOVA performed for Study 3.

Based on human and animal studies by other research laboratories as discussed in the Introduction [14]–[17], we hypothesized that *ad libitum* feeding of dietary pre- and probiotics may have effects on food intake and body weight. To address this hypothesis, mice were given the isocaloric pre- and/or probiotic-containing AIN93G diet for 3–4 weeks in three different studies (Fig. 1). Overall, the pre-or probiotic supplementation had limited effect on the amount of food intake (Fig. 3) and had no effect on body weight (Fig. 4). In the first study of young mice, the food intake of Inulin and Inulin+Bb12 group was slightly higher than the Control group and the difference reached statistical significance at the third week of feeding (Fig. 3A). However, the body weight was not significantly affected by the increase in food intake (Fig. 4A). Furthermore, the increase in food intake by inulin feeding was not observed in the other two studies (Fig. 3B, 3C). Overall, our observation is consistent with the observations in some other long-term pre- and probiotic feeding studies where no dietary effects on food intake or body weight were found [39], [45]. Although epidemiological studies have linked higher fiber intake to lower body weight, it was likely a result of overall lower caloric density in a fiber-rich dietary pattern [46], [47]. As expected from the lack of effect on body weight, neither pre- nor probiotic feeding affected fecal consistency of young or adult mice.

**Figure 5:**
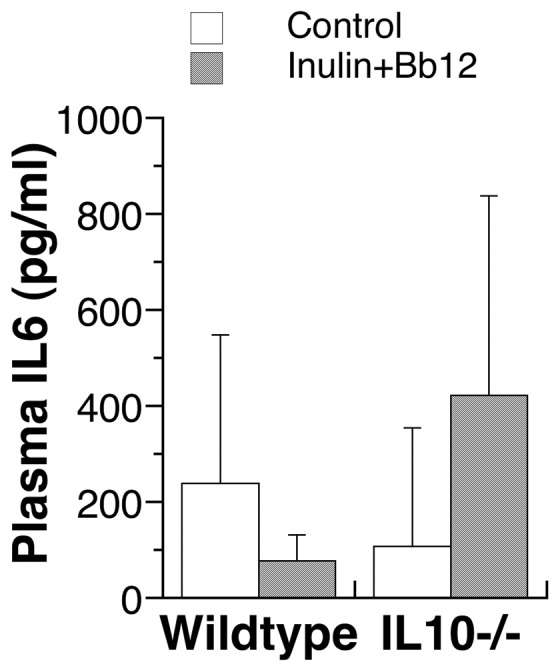
Plasma levels of IL6 in wild type and IL10−/− mice fed pelleted control AIN93G diet or AIN93G diet supplemented with inulin and Bb12. Data are shown as mean±SD. No significant difference was observed for the dietary or genomic effect by Kolmogorov-Smirnov two sample test. N = 5–6.

IL10−/− mice were known to develop intestinal inflammation spontaneously but the condition is strain-, pathogen- and age-dependent [18], [19], [48]. Because we maintained the IL10−/− colony for the study in the specific pathogen-free environment, we predicted that no active disease would be observed in the IL10−/− mice. Indeed, IL10 knockout did not affect the food intake or body weight under Control or Inulin+Bb12 diet (Fig. 3C and 4C). Genotype and diet also had no apparent effects on the fecal consistency; hemoglobin content in the whole blood; and the activity of myeloperoxidase, indicator of white blood cell infiltration in inflammatory bowel diseases [49], in the colon sample (results not shown). In addition, we measured a systemic marker for inflammatory bowel diseases, IL6 [49], [50], and found no genotype or dietary effects (Fig. 5). All these are consistent with the absence of active intestinal diseases and support the general health of our IL10−/− mice.

### The effect of pre- and probiotic feeding on organ weight

**Figure 6:**
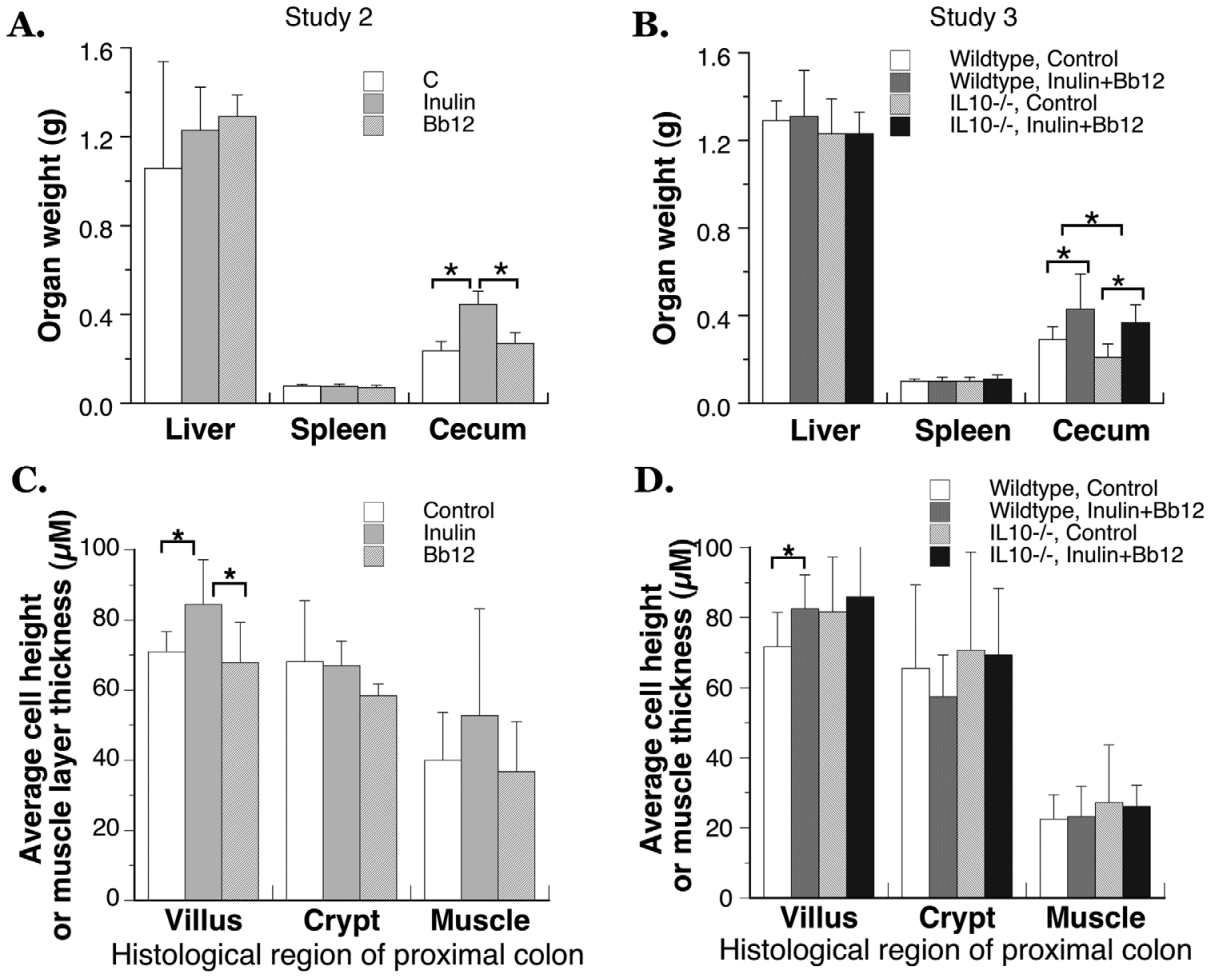
The organ-specific effect of dietary inulin and a lack of effect of probiotic Bb12. (A)(B) No effect on the wet weight of liver and spleen while inulin in the diet significantly increased the wet weight of cecum compared to the control and Bb-12 groups. (C)(D) Inulin in the diet also significantly increased the height of cells at the villus tip in the proximal colon. Data are shown as mean±SD with (A, C) feeding study 2, N = 5-6 in each group; (B, D) feeding study 3, N = 9–14. Some SD bars are too small to be visible. * indicates significant difference at p<0.05. There was no genotype-diet interaction found in any 2-way ANOVA performed for (B)(D).

Weights of liver and spleen were not affected by the pre- and/or probiotic supplementation in either wildtype or IL10-null mice (Fig. 6A–B). This is consistent to the results in Fig. 4 and Fig. 5 where diets showed no effect on body weight or inflammatory indicator. Cecum is a major site of inulin fermentation [4], [5], and higher cecal wet weights (tissue with content) were observed in all mice fed inulin alone or in combination with Bb12 (Fig. 6A–B). Similar cecal weight increase was observed in other fructooligosaccharide-feeding studies [39], [51], [52] and the increase included more cecal content [51], [52]. The organ wet weight increase was specific to cecum, as the weights of liver and spleen were not affected by inulin (Fig. 6A–B). As the site of fermentation, the increase in cecal weight could indicate higher microbial content. This is consistent with the well-established bifidogenic effect of fructooligosaccharide [6]–[10]. Because AIN93G diet has only the poorly fermentable cellulose [53], the growth of probiotics such as Bb12 may not be sustained when only probiotics was included into the diet. Consistent with the prediction, cecal weight was not increased in the Bb12 only group (Fig. 6A). A similar lack of biological effect was observed in other probiotic supplementation studies when probiotics were included in purified diet that contained only poorly fermentable cellulose [37]–[40]. Cecal weight was also increased in IL10−/− mice given Inulin+Bb12 diet (Fig. 6B). This supports the presence of inulin fermentation and an increase in microbial content in the cecum of IL10−/− mice as well. Despite no genotype effect on the body weight and the weights of liver and spleen (Fig. 6B), the cecal weight of IL10−/− mice was significantly less compared to that of wild type mice (Fig. 6B). This genotype effect in cecal weight was found to be independent of the dietary effect as we detected no genotype-diet interaction in two-way ANOVA. Together, the observations in Figs. 3, 4, 5, 6 suggest a mildly compromised intestinal development in IL10 null mice independent of intestinal inflammatory disease. Interestingly, growth-promoting effect of inflammation-related cytokine was reported previously in multiple cell types including the intestinal epithelial cells [54]–[56]. There was only one other animal study that has examined the effect of IL10 knockout in the absence of inflammation. In that study of female IL10−/− mice, less post-pubertal mammary glandular epithelial development was reported in the absence of inflammation [34].

### Effect of the pre- and probiotic feeding on colon

The paraffin sections of proximal colon were examined for dietary and genotype effects. Consistent with the previous publication [57], probiotic and/or prebiotic feeding did not lead to histological abnormality in the colon. Also, as predicted, under our SPF housing condition, there were no signs of enterocolitis in IL10−/− mice and no pathological changes were observed in the proximal colon. No observed intestinal pathology due to IL10 knockout is consistent with no increase in the level of inflammatory markers (Fig. 5). A different study using female IL10−/− mice also observed little histological changes at up to 10 weeks of age [58]. When the cell height of proximal colon was analyzed, dietary intake of inulin, with or without Bb12, was found to increase the height of villus cells in the wild type mice (Fig. 6C–D). Villus cell height has been shown to be an indicator of both epithelial cell differentiation and functional capacity [59]. This dietary effect was not observed in IL10−/− mice (Fig. 6D). Pre- or probiotic supplements had no effect on the heights of crypt or smooth muscle cells (Fig. 6C–D). Dietary Bb12 by itself also did not affect the colonal epithelial cell height (Fig. 6C).

### Effect of the pre- and probiotic feeding on the fecal bile acid pattern

Bile acids secreted from the gallbladder are known to undergo extensive microorganism- and cell-mediated metabolism in the intestine [60]. A representative mass spectrum of bile acids in mouse feces is shown in Fig. S2 and the peaks representing taurine- and sulfate-conjugated bile acids were identified. The structure-based distribution patterns of fecal bile acids are shown in Tables 2–4. Primary bile acid in mice is mainly taurine-conjugated. Taurine conjugation is a signature function of liver [61] because taurine cannot be added to bile acids by intestinal cells or microorganisms. Bile acid hydroxylase activity that removes taurine, on the other hand, is a signature function of intestinal bacteria [28], [62]. Intestinal cells do not have the bile acid hydroxylase activity. Thus, fecal taurine-conjugated bile acid represents the net balance of hepatic synthesis and bacterial removal. It is known that dietary prebiotics do not affect total bile acid pool size [24]. With a constant pool, mice with dietary condition, such as the inulin-containing diet, that promotes the growth of bacteria are expected to have less residual taurine conjugates in the feces. Indeed, results from all three studies support this prediction (Table 2, 3, 4). In addition to being bifidogenic based on the fecal analysis [6]–[10], inulin ingestion was found to modify the mucosa-associated microbiota of the human large intestine [7]. Because bacterial species- and strain-difference in the bile acid hydroxylase activity was reported [62], it is possible that the alteration in the bacterial population may have also contributed to more hydrolase activity. All three studies here had 3–4 weeks of the same level of inulin intake, future studies are needed to determine the dose and duration of inulin intake that are needed for the effect. The application of fecal bile acid profiling to demonstrate an increased fermentation was nevertheless novel. The possibility of performing repeated fecal sampling on the same subject; along with the stability of fecal bile acids during storage make the profiling approach suitable for the assessment of other potential prebiotics in healthy subjects.

**Table 2:** Effect of 3-week feeding of dietary inulin and probiotic, Bb12, on the relative peak height and proportion of different classes of fecal bile acids and cholesterol sulfate of young mice^a^.

	Control diet	Inulin	Bb12	Inulin+Bb12
	N			
	4	4	4	3
Relative peak height of bile acid (% relative to peak at m/z 407)				
1. Taurine conjugate^b^	3.5±3.3	1.0±1.4	2.3±1.8	0.15±0.26
2. Unconjugated^c^	177.0±15.6	165.1±8.9	185.0±32.4	138.7±5.4
3. Sulfate conjugates^d^	11.6±19.4	13.8±10.9	9.3±11.8	12.6±11.0
Total bile acids^e^	192.1±27.3	179.8±6.9	196.7±35.3	151.5±6.7
Cholesterol sulfate^f^	13.2±4.8	12.3±1.7	12.5±1.9	10.5±5.7
Relative peak height to total bile acids (% relative to (1+2+3))				
Taurine conjugates/total^g,h^	**2.0±2.1**	**0.55±0.75**	**1.2±1.0**	**0.097±0.168**
Sulfate conjugates/total^i,j^	**5.2±8.3**	**7.6±5.9**	**4.4±5.4**	**8.1±6.9**
Cholesterol sulfate/total^k^	6.8±2.1	6.8±0.9	6.4±0.9	6.8±3.

a Values presented are means±SD. The absolute peak height of m/z 407 was 106±0 mm for all four groups. ^b^Peak height of m/z 496 relative to the peak height of m/z 407. ^c^Sum of peak height of m/z 375, 389, 391, 405, 407, 423 relative to the peak height of m/z 407. ^d^Sum of peak height of m/z 471, 485, 487, 493, 507, 509 relative to the peak height of m/z 407. ^e^Sum of peak height of taurine conjugate, unconjugated and sulfate conjugates (1+2+3) relative to the peak height of m/z 407. ^f^Peak height of m/z 465 relative to the peak height of m/z 407. ^g^Value of Taurine conjugate divided by Total bile acids. ^h^Fecal taurine conjugates in the two groups with dietary inulin was significantly less (p<0.05) than that in the two groups without dietary inulin by Kolmogorov-Smirnov two sample test.^ i^Value of Sulfate conjugates divided by Total bile acids. ^j^Significantly (p<0.05) more sulfate conjugates than taurine conjugates in the feces by Kolmogorov-Smirnov two sample test. ^k^Value of Cholesterol sulfate divided by Total bile acids.

**Table 3:** Effect of 4-week feeding of dietary inulin and Bb12 on the relative peak height and proportion of different classes of fecal bile acids and cholesterol sulfate in adult mice^a^.

	Control diet	Inulin	Bb12
	N		
	6	6	6
Relative peak height of bile acid (% relative to peak at m/z 407)			
1. Taurine conjugate^b^	4.9±2.9	0.5±1.3	7.1±7.4
2. Unconjugated^c^	161.9±18.6	177.6±21.3	183.2±14.2
3. Sulfate conjugates^d^	35.2±27.1	43.6±22.3	50.6±50.3
Total bile acids^e^	202.0±33.1	221.7±38.3	239.6±48.4
Cholesterol sulfate^f^	18.0±11.7	28.3±17.5	44.6±44.6
Relative peak height to total bile acids (% relative to (1+2+3))			
Taurine conjugates/total^g^	**2.6±1.8**	0.27±0.66*	**2.7±3.3**
Sulfate conjugates/total^h. i^	**16.3±10.7**	**18.9±7.1**	**18.8±14.1**
Cholesterol sulfate/total^j^	8.5±4.5	13.9±10.9	16.9±13.0

a Values presented are means±SD. The absolute peak height of m/z 407 was 101±11, 106±0, and 102±11 mm for control, inulin and Bb12 group, individually. *Fecal taurine conjugates in the inulin group was significantly less (p<0.05) than that in the control group and Bb12 group by Kolmogorov-Smirnov two sample test. ^b,c,d,e,f,g^Same as legends in Table 2.^ h^Value of Sulfate conjugates divided by Total bile acids. ^i^Sulfate conjugates was significantly more (p<0.05) than taurine conjugates in the feces by Kolmogorov-Smirnov two sample test. ^j^Value of Cholesterol sulfate divided by Total bile acids.

**Table 4:** Effect of 4-week feeding of dietary inulin and Bb12 in combination on the relative peak height and proportion of different classes of fecal bile acids and cholesterol sulfate in adult wild type and IL10−/− mice^a^.

	Wild type		IL10−/−			
	Control diet	Inulin+Bb12	Control diet		Inulin+Bb12	
	N					
	6	6	7	7		
Relative peak height of bile acid (% relative to peak at m/z 407)						
1. Taurine conjugate^b^	18.5±16.6	3.9±4.2	14.3±9.2		10.4±16.6	
2. Unconjugated^c^	190.6±29.1	179±39.2	184.2±20.4		184.2±33.4	
3. Sulfate conjugates^d^	34.1±13.6	60.5±35.0	26.4±16.3			32.4±26.2
Total bile acids^e^	243.2±35.7	244.6±69.9	224.9±28.4			226.9±56.1
Cholesterol sulfate^f^	29.5±13.0	25.2±27.2	37.9±23.3			27.5±14.7
Relative peak height to total bile acids (% relative to (1+2+3))						
Taurine conjugates/total^g,h^	**7.1**±**5.4**	**2.0**±**1.8**	**6.4**±**3.8**			**4.3**±**6.0**
Sulfate conjugates/total^i,j^	**14.2**±**5.8**	**23.7**±**7.8**	**11.5**±**6.4**			**13.1**±**9.4**
Cholesterol sulfate/total^k^	12.3±5.5	9.0±6.3	16.4±8.5			11.6±4.9

a Values presented are means±SD. The absolute peak height of m/z 407 was 97±22, 99±15, 103±9 and 106±0 mm for wild type control, wild type inulin+Bb12, IL10−/− control, and IL10−/− inulin+Bb12 group, individually. ^b,c,d,e,f,g^Same as legends in Table 2. ^h^ Fecal taurine conjugates in the inulin+Bb12 diet group was significantly less (p<0.05) than that in the Control diet group by Kolmogorov-Smirnov two sample test. ^i^Value of Sulfate conjugates divided by Total bile acids. ^j^Significantly more (p<0.05) sulfate conjugates than taurine conjugates in the feces by Kolmogorov-Smirnov two sample test. ^k^Value of Cholesterol sulfate divided by Total bile acids.

We did not observe any effect of probiotic-feeding by itself on fecal bile acids (Table 2–3). A lack of effect of probiotics on taurine conjugates is consistent with the lack of effect on cecal weight. Without the fermentable fiber intake, probiotics cannot thrive. The overall lack of effect of Bb12 diet in all measurements (Fig. 3, 4, 5, Table 2, 3, 4) was not due to a loss of Bb12 during diet storage. Our mouse diets were validated for Bb12 viability (Fig. 2). Probiotic studies that have reported biological activities invariably used commercial plant-based rodent chow, which has significant amount of fermentable fiber. Probiotic supplementation by itself in purified diet without fermentable fiber was found to be biologically ineffective in other animal studies as well [37]–[40].

Primary bile acids contained little sulfate conjugates. Sulfate conjugation of secondary bile acids happened after intestinal bacteria-mediated taurine deconjugation and additional dehydroxylation and dehydrogenation. These secondary sulfate conjugates were proposed to be products of large intestinal cellular sulfotransferase [29] and were measured in our studies. Ingesting diets containing inulin led to a trend of increase in the percentage of sulfate conjugates in all three studies (Tables 2, 3, 4) but none of the observation was statistically significant by the nonparametric Kolmogorov – Smirnov two sample test. There was also no genotype difference in the level of sulfate conjugates. Among all studies, young mice (Table 2) had a lower proportion of bile acids in the form of sulfate conjugates than did adult mice (Table 3–4), which is consistent with the known postnatal maturation of intestinal function [63]. Overall, we observed a significant negative correlation between the percentage of fecal taurine conjugate and that of fecal sulfate conjugate (r = −0.39, p<0.05) (Tables 2, 3, 4). This negative correlation further supports that sulfate conjugates were produced after the deconjugation of primary taurine conjugate.

Cholesterol sulfate was detected in the feces as well (Tables 2, 3, 4). It is made throughout the gastrointestinal tract [64] and found in the feces primarily as a result of cell shedding [65]. Dietary inulin and/or Bb12 did not induce a consistent trend of change in cholesterol sulfate secretion (Tables 2, 3, 4). There was also no genomic effect on fecal cholesterol sulfate (Table 4). In summary, fecal bile acid profiling potentially can be a useful approach to grasp the effect of diet on the *in vivo* environment of large intestine but more studies are needed to understand the power and limit of this approach.

IL10 knockout led to a smaller cecum (Fig. 6B) despite normal growth and free of inflammation. Fiber fermentation was apparent in IL10−/− mice judging by the similar extent of increase in cecum weight compared to the wild type mice (Fig. 6B). However, the colonic response to Inulin+Bb12 diet was less in IL10−/− mice. The villus cell height was not affected by the diet in IL10−/− mice (Fig. 6D). The increase in fecal sulfate-conjugated bile acids may also be less in IL10−/− mice when given Inulin+Bb12 diet (Table 4). With the proposed three-way talk between the immune system, commensal bacteria and intestinal epithelial cells [21], [22], there are two possible explanations for the compromised responses: one is an effect of IL10 knockout on the constituents of microbiota and the second one is the resistance of colon epithelial cells of IL10−/− mice to the dietary/microbial influence. Future studies are needed to dissect out these two potential mechanisms.

## Conclusions

Using purified isocaloric diets, we examined the biological effects of dietary pre- and probiotics separately and in combination in wildtype and IL10-null mice. In Aim 1, food intake, body weight and the weight of liver and spleen were not affected by isocaloric dietary supplementation of pre- and/or probiotics in young and adult mice, suggesting that caloric density, rather than pre- or probiotics *per se*, is a main determinant of body weight. Similar to published observation, inulin-supplemented mice showed increased cecal weight, which is consistent with an increased growth of commensal bacteria. In Aim 2, alterations in fecal bile acid pattern were consistently observed after the dietary intake of prebiotics in the absence or presence of probiotics but the change did not impact on the intestinal fluid balance. Nevertheless, our findings support that fecal bile acid profiling, especially a reduction of taurine conjugates in the feces, may be a useful biomarker for the intake of prebiotics in mice and potentially in human as well. Dietary probiotic Bb12 alone affected neither cecal weight nor the fecal bile pattern at the dose used in this study. In the subpart of Aim 1 and Aim 2, IL10-null mice showed a similar trend of responses to pre- and probiotic supplementation as the wildtype mice. However, a slightly smaller cecum was observed in IL10-null mice in the absence of inflammation, which is consistent to the known growth-promoting effect of inflammation-related cytokine reported previously.

## Supporting Information

Figure S1 **Partial image of a paraffin section of proximal colon stained with hematoxylin and eosin and used for the histological analysis shown in** **Fig. 6C and 6D** **.** Twelve tile images were first acquired by Zeiss AxioImager microscope with 20× objective and an integrated AxioCam Hrc digital camera. Mosaic function in AxioVision 4.8 software was used to control the collection of the twelve tile images and the eventual merging of them to the image shown. Representative regions used for the quantitative analysis of cell types shown in Figure 6C and 6D are labeled.
(TIF)

Figure S2 **Representative mass spectrum of bile acids in mouse feces.** Mass-to-charge ratios (m/z) of peaks are shown. Peak height represents relative abundance. Peaks at m/z 375, 389, 391, 405, 407 and 423 represent unconjugated bile acids/salts. Peak at m/z 407, a trihydroxy C24 bile acid, is also the reference peak because of its abundance and its peak height was set as 100%. The peak at m/z 496 represents monohydroxy, mono-oxo C24 taurine-conjugated bile acid. Sulfate conjugated bile acids detected including the parent and sodium salt forms of monohydroxy, mono-oxo; dihydroxy; and trihydroxy C24 sulfate conjugates. *: Peak of taurine-conjugated bile acid.*: Peaks of sulfate-conjugated bile acids/salts.
(TIF)
