# Supplementary figures and images for: The Effect of Dietary Prebiotics and Probiotics on Body Weight, Large Intestine Indices, and Fecal Bile Acid Profile in Wild Type and IL10−/− Mice
Source: PLoS One. 2013 Mar 21;8(3):e60270. doi: 10.1371/journal.pone.0060270 (PMC3605333; doi:10.1371/journal.pone.0060270)

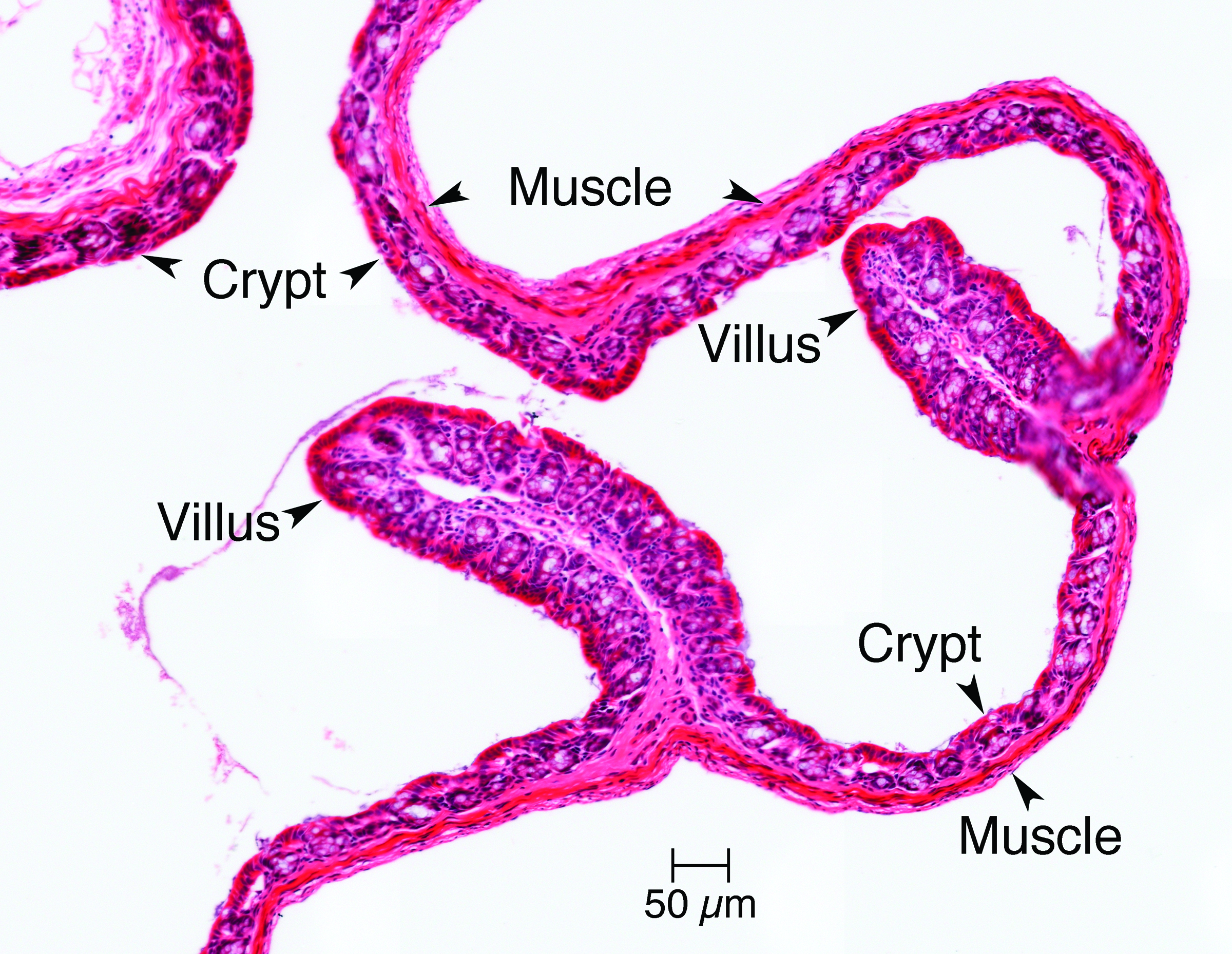

Supplement: Figure S1 — Partial image of a paraffin section of proximal colon stained with hematoxylin and eosin and used for the histological analysis shown in Fig. 6C and 6D . Twelve tile images were first acquired by Zeiss AxioImager microscope with 20× objective and an integrated AxioCam Hrc digital camera. Mosaic function in AxioVision 4.8 software was used to control the collection of the twelve tile images and the eventual merging of them to the image shown. Representative regions used for the quantitative analysis of cell types shown in Figure 6C and 6D are labeled. (TIF) [file pone.0060270.s001.tif]

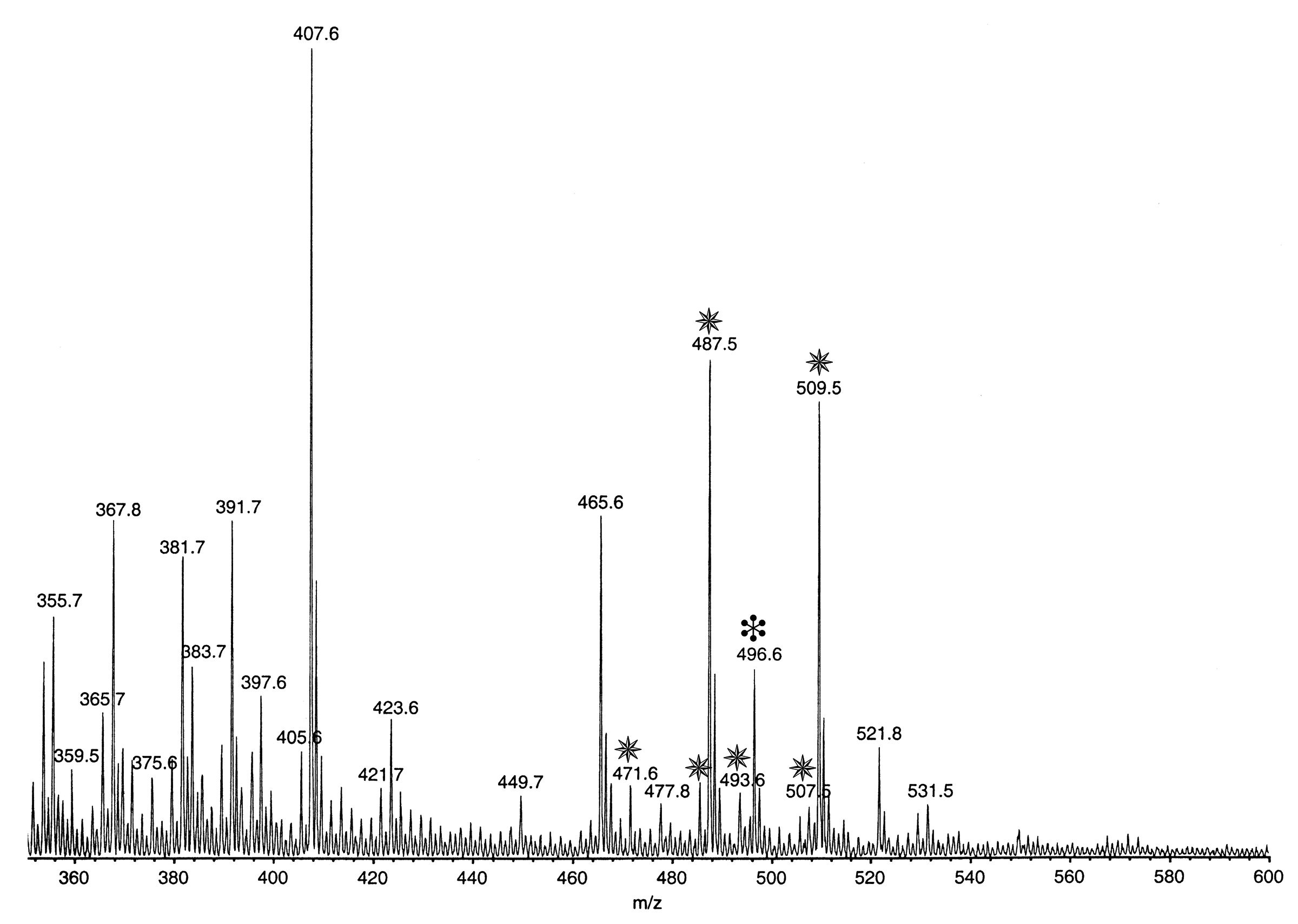

Supplement: Figure S2 — Representative mass spectrum of bile acids in mouse feces. Mass-to-charge ratios (m/z) of peaks are shown. Peak height represents relative abundance. Peaks at m/z 375, 389, 391, 405, 407 and 423 represent unconjugated bile acids/salts. Peak at m/z 407, a trihydroxy C24 bile acid, is also the reference peak because of its abundance and its peak height was set as 100%. The peak at m/z 496 represents monohydroxy, mono-oxo C24 taurine-conjugated bile acid. Sulfate conjugated bile acids detected including the parent and sodium salt forms of monohydroxy, mono-oxo; dihydroxy; and trihydroxy C24 sulfate conjugates. *: Peak of taurine-conjugated bile acid.*: Peaks of sulfate-conjugated bile acids/salts. (TIF) [file pone.0060270.s002.tif]
